# *Salmonella enterica* Serovar Enteritidis, England and Wales, 1945–2011

**DOI:** 10.3201/eid2007.121850

**Published:** 2014-07

**Authors:** Christopher R. Lane, Susan LeBaigue, Oluwaseun B. Esan, Adedoyin A. Awofisyo, Natalie L. Adams, Ian S.T. Fisher, Kathie A. Grant, Tansy M. Peters, Lesley Larkin, Robert H. Davies, Goutam K. Adak

**Affiliations:** Public Health England, London, UK (C.R. Lane, S. LeBaigue, O.B. Esan, A.A. Awofisayo, N.L. Adams, I.S.T. Fisher, K.A. Grant, T.M. Peters, G.K. Adak);; Animal Health and Veterinary Laboratories Agency, London (L. Larkin, R.H. Davies)

**Keywords:** Salmonella enterica serovar Enteritidis, Salmonella enterica, salmonellosis, eggs, chickens, chicken meat, gastroenteritis, outbreaks, epidemiologic surveillance, England, Wales, salmonellae, enteric infections, bacteria, intestinal infections, epidemic

## Abstract

In England and Wales, the emergence of *Salmonella enterica* serovar Enteritidis resulted in the largest and most persistent epidemic of foodborne infection attributable to a single subtype of any pathogen since systematic national microbiological surveillance was established. We reviewed 67 years of surveillance data to examine the features, underlying causes, and overall effects of *S. enterica* ser. Enteritidis. The epidemic was associated with the consumption of contaminated chicken meat and eggs, and a decline in the number of infections began after the adoption of vaccination and other measures in production and distribution of chicken meat and eggs. We estimate that >525,000 persons became ill during the course of the epidemic, which caused a total of 6,750,000 days of illness, 27,000 hospitalizations, and 2,000 deaths. Measures undertaken to control the epidemic have resulted in a major reduction in foodborne disease in England and Wales.

## Abstract 2

A focus on eliminating phage type 4 in egg and poultry production has greatly reduced foodborne disease among humans.

A pandemic of *Salmonella enterica* serovar Enteritidis infection was recognized by epidemiologists in the United States in the late 1970s; a 6-fold rise in these infections was observed in northeastern United States during 1976–1986 (*1*). A review of outbreak investigations revealed that 27 (77%) of 35 outbreaks were associated with the consumption of foods containing grade A eggs (*1*). The most commonly reported phage types were SE8, SE13, and SE13a. In 1990, the World Health Organization reviewed *Salmonella* surveillance data for 1979–1987 and found that isolation rates for *S. enterica* ser. Enteritidis had increased in 24 of the 35 nations that provided data. Increases were recorded in countries from every continent except Asia (*2*). Evidence from outbreak investigations in Spain, Hungary, France, Norway, and the United States implicated eggs (*3*). Microbiologicical investigations conducted in the United Kingdom also showed the presence of phage type SE4 in chicken meat (*4*) and raw shell eggs (*5*,*6*). In 1988, the UK Public Health Laboratory Service Communicable Disease Surveillance Centre conducted a case–control study of primary sporadic SE4 infections in England. The investigators demonstrated associations between human infection and the consumption of chicken and raw egg dishes (*7*). We reviewed national surveillance and research data to examine the factors underlying the epidemic of *S. enterica* ser. Enteritidis and to estimate its overall impact on the population of England and Wales.

## Methods

### Surveillance of *S. enterica* Infections and Other Intestinal Diseases in England and Wales

Systematic national surveillance of laboratory-confirmed salmonellosis in humans in England and Wales has been in continuous operation since 1945. Diagnostic laboratories refer all *Salmonella* isolates to the national reference laboratory for confirmation and characterization, and data on all first confirmations are entered into a national surveillance database (*8*).

We extracted data from this database to provide annual totals for human infection with *S. enterica* by serotype and phage type. Multipliers derived from previous studies (*9*–*11*) were applied to the number of laboratory reports received to produce estimates of the numbers of community cases, days of illness, hospitalizations, hospital bed-days occupied, and deaths for 1982–1987, 1988–1998, and 1999–2011 that were attributable to SE4. Multipliers published in 1996 (*9*) were used for the emergence and epidemic stages and those from 2008 (*10*) for the decline stage.

In addition, local health protection units return standardized data (i.e., etiology, outbreak location, morbidity/mortality rates, vehicles of infection, and evidence of association) on all detected general outbreaks of infectious intestinal diseases to national surveillance (*12*). These data are also stored in a dedicated database.

### Surveillance of *S. enterica* in Poultry

Data on *Salmonella* spp. in poultry in Great Britain (England, Wales, and Scotland) are reported by the Animal Health and Veterinary Laboratory Agency (*13*). A *Salmonella* incident is defined as the first isolation of a given serovar from a particular animal, group of animals, or their environment on a single premises within a defined period (usually 30 days) (*13*).

### Data Analyses

Data were abstracted from the national surveillance databases described above. Descriptive analyses were done in Microsoft Excel 2007 (Microsoft Corporation, Redmond, WA, USA); 95% CIs of the estimates of the burden of disease in the community were calculated from the upper and lower confidence limits reported in previous studies (*9**,**10*). All statistical analyses were performed by using Stata version 12 (StataCorp LP, College Station, TX, USA).

## Results

### Trends in Human Salmonellosis in England and Wales

**Figure 1:**
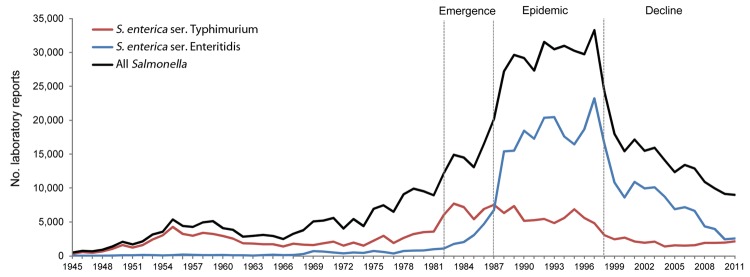
Laboratory reporting of *Salmonella enterica* infections in England and Wales, 1945–2011. Emergence stage, 1982–1987; epidemic stage, 1988–1998; decline stage, 1999–2011. Ser., serovar.

Figure 1 shows the contribution of *S. enterica* ser. Enteritidis to the overall scope of human salmonellosis in England and Wales during 1945–2011. During this period, >740,000 laboratory reports of *S. enterica* infection were received; almost 330,000 (43%) were for *S. enterica* ser. Enteritidis. The reporting patterns show that the epidemiology of this pathogen can be divided into 4 stages: pre-epidemic (1945–1981); emergence (1982–1987); epidemic (1988–1998); and decline (1999 onwards).

The surveillance trends for *S. enterica* for the years 1945–1981 mainly reflect the reporting patterns for serotype Typhimurium; for most of this period, this serotype was the most commonly reported, whereas serotype Enteritidis accounted for <10% of cases of salmonellosis in all but 5 of the 37 years of the pre-epidemic stage. During the emergence stage, the percentage of salmonellosis cases caused by serotype Enteritidis rose from 9% (1,099 reports) to 33% (6,746 reports). In 1988, serotype Enteritidis supplanted serotype Typhimurium as the most commonly reported serotype.

*S. enterica* ser. Enteritidis accounted for more than half of all salmonellosis cases for all of the epidemic stage (1988–1998). In 1997, reporting of serotype Enteritidis accounted for 70% (23,231 reports) of all salmonellosis cases. During the decline stage, the share of salmonellosis attributable to serotype Enteritidis fell from 60% (10,827 reports) to 28% (2,566 reports in 2011). Despite its sharp decline during the final years of the surveillance period, however, reporting of serotype Enteritidis has remained above the levels observed during the pre-epidemic stage.

### Surveillance of *S. enterica* ser. Enteritidis, 1982–2011

**Figure 2:**
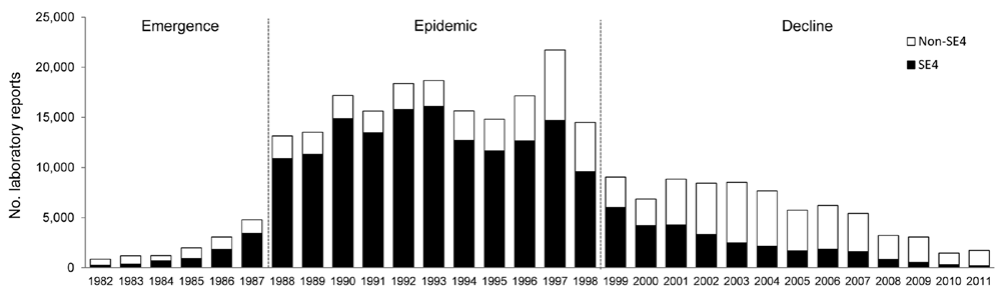
Laboratory reporting of indigenously acquired *Salmonella* *enterica* serovar Enteritidis infections in England and Wales, 1982–2011. Emergence stage, 1982–1987; epidemic stage, 1988–1998; decline stage, 1999–2011. SE4, *S.* *enterica* ser. Enteritidis phage type 4.

We examined trends in the reporting of *S. enterica* ser. Enteritidis during 1982–2011 in more detail. During this period, 312,719 laboratory reports for serotype Enteritidis were received. After reports of travel-associated infection were excluded, 269,779 reports remained. In 1982 and 1983, SE8 was the most commonly identified phage type, accounting for ≈60% of all cases. Indigenously acquired infection with SE4 was reported at a crude rate of 0.5 cases/100,000 population in 1982. However, in 1984, SE4 became the dominant phage type, contributing 57% of all indigenously acquired infections (crude rate 1.4 cases/100,000 population). Figure 2 shows that the emergence stage marked an accelerating rise in indigenously acquired SE4 infection in England and Wales. During this period, the incidence of indigenously acquired SE4 infection was sustained at or above a crude rate of 30 cases/100,000 population. The reporting of SE4 infections reached its peak in 1993 at 16,127 laboratory reports (i.e., 86% of all indigenously acquired *S. enterica* ser. Enteritidis infections).

The decline stage was characterized by absolute and relative reductions in the contribution of SE4 to the overall scope of *S. enterica* ser. Enteritidis infection. By 2011, the crude rate of reporting had fallen to 0.4 cases/100,000 population. This stage also represents a period when other phage types came into prominence. Even so, for every year from 1984 to 2001, SE4 accounted for more than half of all indigenously acquired *S. enterica* ser. Enteritidis infections.

During its emergence, 7,481 reports of indigenous SE4 infection were received. This compares with 143,767 reports received during the epidemic stage and 29,522 during the decline. Estimates for the burden of indigenous disease attributable to SE4 infection during the emergence, epidemic, and decline stages are shown in the Table.

**Table:** Estimated rates of disease attributable to *Salmonella enterica* serovar Enteritidis phage type 4 during 3 periods, England and Wales, 1982–2011

Stage	No. laboratory-confirmed cases	No. community cases (95% CI)	No. days of illness	No. hospital admissions	No. hospital bed-days	No. deaths
Emergence, 1982–1987	7,481	16,458 (8,379–71,817)	270,000	1,000	6,000	90
Epidemic, 1988–1998*	143,767	374,516 (161,019–1,380,163)	5,000,000	21,000	122,000	1,630
Decline, 1999–2011†	29,522	135,801 (41,331–661,292)	1,300,000	5,000	30,000	410
Total	180,770	526,766	6,570,000	27,000	158,000	2,130

*Multiplier from (*9*). †Multiplier from (*10*).

Demographically, we found no significant regional or gender differences in the reporting rates for SE4 infection. Children <14 years of age consistently accounted for one quarter of all cases (crude rates: 1982, 0.4 cases/100,000 population; 1984, 1.1 cases/100,000; 1992, 45 cases/100,000; 2011, 0.6 cases/100,000).

### Surveillance of General Outbreaks of Infection in England and Wales, 1992–2011

Standardized surveillance reports were returned for 2,667 general outbreaks of foodborne infection in England and Wales during 1992–2011. *S. enterica* was the causative agent in 1,195 (45%) outbreaks; 914 (34%) cases were attributable to *S. enterica* ser. Enteritidis, of which 585 (22%) were attributable to SE4. In the portion of the *S. enterica* ser. Enteritidis epidemic stage during which general outbreak surveillance was in operation (1992–1998), SE4 infections accounted for 474 (30%) of the 1,576 outbreak reports received, compared with 7% for other *S. enterica* ser. Enteritidis (non-SE4). However, during the decline stage, the proportion of foodborne outbreaks caused by SE4 infections fell to 10% (111/1,082), and during the last 5 years of surveillance (2007–2011), SE4 accounted for only 3% of outbreaks (10/330).

**Figure 3:**
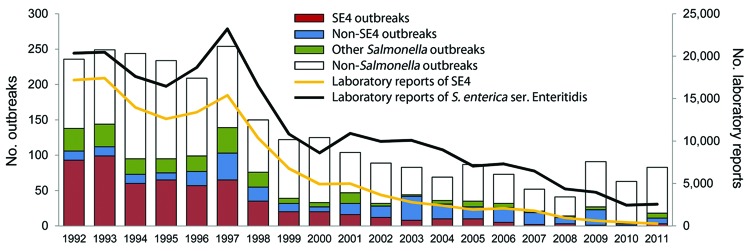
Trends in the pathogens associated with general outbreaks of foodborne infection in England and Wales, 1992–2011. SE4, *Salmonella* *enterica* serovar Enteritidis phage type 4.

During 1992–2011, the trends in the reporting of foodborne outbreaks in England and Wales were partially driven by outbreaks of SE4 infections (Figure 3). During 1992–2011, a total of 9% (12,647/133,959) of all SE4 laboratory reports received were linked to general outbreaks. By 2011, the numbers of SE4 laboratory reports and general outbreaks had fallen to 1% of the 1992 reporting levels.

**Figure 4:**
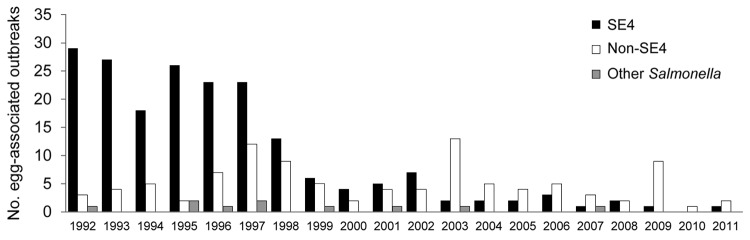
Trends in the reporting of general outbreaks of salmonellosis associated with the consumption of eggs in England and Wales 1992–2011. SE4, *Salmonella* *enterica* serovar Enteritidis phage type 4.

Vehicles of infection were identified in 471 (80%) of 585 SE4 outbreaks reported during 1992–2011. Chicken meat accounted for 76 (16%) outbreaks, but chicken-associated outbreaks of SE4 declined sharply during the surveillance period. During 1992–1993, a total of 31 (16%) of 192 SE4 outbreaks were attributable to chicken meat, but during 1994, the proportion of SE4 infections attributable to chicken meat fell to 10% (4/39), where it remained through 2011. By contrast, 195 (41%) of the SE4 outbreaks were attributable to egg consumption. During the epidemic stage, SE4 accounted for 159 (79%) of 201 egg-associated *S. enterica* ser. Enteritidis outbreaks (Figure 4). The decline stage was marked by sharp falls in the number and proportion (36/95 [38%]) of egg-associated *S. enterica* ser. Enteritidis outbreaks attributable to SE4. Only 5 egg-associated outbreaks of SE4 infection were reported during 2007–2011. By contrast, the contribution of non-SE4 isolates rose from 21% (42/201) during 1992–1998 to 62% (59/95) during 1999–2011.

Lightly cooked desserts were the most commonly reported egg-based vehicles of infection implicated in *S. enterica* ser. Enteritidis outbreaks during the epidemic stage. This group excludes cakes but includes custard-based desserts such as tiramisu and zabaglione; mousses; meringues; and custom-made ice creams and sorbets. This category accounted for 109 (54%) of the 201 egg-associated *S. enterica* ser. Enteritidis outbreaks reported during 1992–1998; of these outbreaks, 80 (40%) were attributable to SE4. In the 13 following years, the proportion of egg-associated outbreaks associated with these desserts fell to 33% (31/95); half of these (16) were caused by SE4.

Lightly cooked/uncooked sauces made from raw eggs (e.g., hollandaise sauce, mayonnaise) were implicated in 24 (12%) of the 201 egg-associated *S. enterica* ser. Enteritidis outbreaks during the epidemic stage; 22 (92%) of these were caused by SE4. Thirteen sauce-associated outbreaks were reported in the following 13 years; 3 (23%) were caused by SE4.

In contrast to other food vehicles, the number of outbreaks associated with simple egg dishes (i.e., fried eggs, boiled eggs, scrambled eggs, omelets, egg fried rice) increased during the decline stage. During 1992–1998, simple egg dishes were implicated in 51 (25%) of 201 outbreaks; the number rose to 49 (52%) of 95 outbreaks during 1999–2011. The proportion of outbreaks associated with simple egg dishes that were attributable to non-SE4 rose from 12% (6/51) during the epidemic stage to 67% (33/49) during the decline stage.

Only 7 outbreaks linked to eggs served in Chinese restaurants were reported during 1992–1998; all were caused by SE4. A total of 21 outbreaks linked to Chinese restaurants were reported during 1999–2011, and 4 were caused by SE4. The dish most commonly implicated was egg fried rice (22/28 outbreaks [79%]).

### Surveillance of *S. enterica* Infection in Livestock

We found few national surveillance reports of *S. enterica* ser. Enteritidis in nonpoultry livestock. For the few incidents in which the pathogen was identified in cattle, sheep, pigs, and turkeys, SE4 was the predominant phage type isolated.

**Figure 5:**
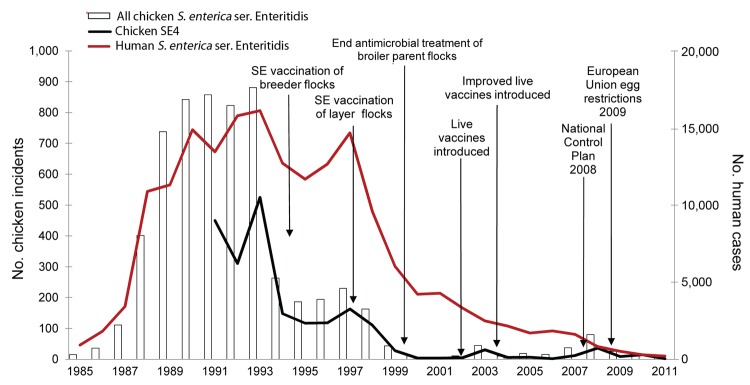
Trends in the reporting of incidents of *Salmonella enterica* in chickens in Great Britain versus laboratory reporting of human *S.* *enterica* serovar Enteritidis infection, England and Wales, 1985–2011. SE4, *S.* *enterica* ser. Enteritidis phage type 4.

We compared trends in national surveillance data for *S. enterica* in chickens in Great Britain during 1985–2011(Figure 5) with those for human infection. Post-1991 data showed that a high proportion of the outbreaks from 1985–1990 were likely to be the result of SE4 infection.

The trends in the reporting of *S. enterica* ser. Enteritidis in chickens and cases of human infection were in general agreement during the emergence stage and the first 6 years of the human epidemic stage (1988–1993). The contribution of *S. enterica* ser. Enteritidis to reported incidents of salmonellosis rose from 3% (15/553) in 1985 to 66% (881/1,342) in 1993, the year in which vaccination of breeder chicken flocks against this pathogen was introduced. A 2-stage decline followed; the first stage was marked by a 70% (618/881) decrease in reports of *S. enterica* ser. Enteritidis infections in chickens during the 1994 calendar year, corresponding with wide uptake of vaccination among breeding flocks. A plateau in reporting was then observed for the remainder of the human epidemic (1994–1998); case levels were maintained at 20%–30% of the 1993 value.

The second stage of decline followed the introduction and subsequent extension of the vaccination program (R.H. Davies, pers. comm.), enhanced farm hygiene, and management standards implemented through a farm assurance scheme for major egg layer flocks in 1997 (*14*). This decline lasted for 2 years. Since 1999, incident reporting has remained below 5% of 1993 levels for all but 2 of 12 years. Reporting has shown an ongoing decline that corresponds with extension of vaccination and improved control measures to smaller-scale egg producers; industry preparations for the implementation of the *Salmonella* National Control Programme in commercial laying chicken flocks in 2008; and application of harmonized European Union–wide restrictions on sale of fresh eggs from flocks infected with *S. enterica* ser. Enteritidis or Typhimurium, which began in 2009 (Figure 5). In 2001, attenuated vaccines were replaced by live vaccines, and in 2003, improved *S. enterica* ser. Gallinarum rough mutant 9R auxotrophic live vaccines were adopted.

During the 27-year period, *S. enterica* ser. Enteritidis accounted for 24% (6,074/25,049) of reported *S. enterica* incidents in chickens. However, 94% (5,690/6,074) of these incidents were reported during 1987–1998, the height of the human epidemic.

## Discussion

Our examination of almost 7 decades of national surveillance data leads us to the conclusion that the emergence of *S. enterica* ser. Enteritidis infection in 1982 resulted in the largest, most persistent epidemic of foodborne infection attributable to a single subtype of any pathogen since systematic national microbiologic surveillance of disease was established in England and Wales. The national *Salmonella* surveillance dataset provides an uninterrupted, 67-year record of the epidemiology of human *S. enterica* infection in England and Wales. Our analyses of the serotype and phage typing dataset enabled us to examine the size and duration of epidemics of foodborne infection caused by subtypes belonging to a range of serovars of *S. enterica* that have occurred since 1945. These epidemics included several sustained, high-impact outbreaks: *S. enterica* ser. Typhimurium during 1949–1961(*15*); *S. enterica* ser. Agona during the late 1960s/early 1970s; *S. enterica* ser. Hadar during the late 1970s; *S. enterica* ser. Typhimurium DT204 during the early 1980s (*14*); and *S. enterica* ser. Typhimurium DT104 during the 1990s (*16*).

The *S. enterica* ser. Typhimurium epidemic of the 1950s gave rise to ≈20,000 excess laboratory reports in 12 years, a mean of 1,667 per year. By comparison, our estimates indicate that the SE4 epidemic gave rise to an excess of ≈160,000 laboratory reports of indigenous infection over 30 years, a mean of 5,333 per year.

The underlying causes that lay behind the rise and fall of earlier epidemics of salmonellosis are poorly understood (*15*). The scale and geographic reach of the rise of *S. enterica* ser. Enteritidis were recognized at an early stage, which led to the development of concerted national and international initiatives. These efforts have enabled scientists to gain better insight into the factors that mediated the course of what is now recognized as a sustained and continuing foodborne pandemic. The work of scientists from many countries has shown that *S. enterica* ser. Enteritidis emerged and quickly became established in much of the global poultry flock (*2*). An ecologic niche may have been created after the introduction of eradication programs targeted against *S. enterica* serovars Pullorum and Gallinarum and as a result of international trade in infected breeding stock (*17*), before the importance of *S. enterica* ser. Enteritidis infection was recognized and minimal monitoring was put in place (*18**,**19*).

Surveillance data demonstrate that the rates of human *S. enterica* ser. Enteritidis infection in England and Wales remained high during 1988–1998 despite national guidance aimed at the public and industry (*20*). From 1997 to 2011, disease incidence decreased 99%. This decrease cannot be explained by changes in the performance of surveillance resulting from the behavior of patients, clinicians, or laboratories. The results of 2 studies of intestinal disease (*9**,**10*) demonstrate that relatively small changes in the ascertainment of salmonellosis by laboratory report surveillance occurred during this period.

Comparison of trends in the reporting of incidents in the chicken flock in Great Britain with human surveillance data showed that the rise in human *S. enterica* ser. Enteritidis infection matched the rise in disease in chicken farms. Reporting of incidents in chickens started to decrease in 1994, after the introduction of a voluntary national vaccination and flock hygiene program targeted at breeder chicken flocks (*21*) (Figure 5). The vaccine was not specific to SE4. Although the program was not mandatory, anecdotal stakeholder information indicates that it was adopted by a large proportion of the industry. The reduction of reported infection in chickens appears to have had a limited effect on the trend in human infection as measured by laboratory report surveillance (Figure 5). However, the reporting of outbreaks of *S. enterica* ser. Enteritidis associated with the consumption of chicken also showed a sharp decline dating from 1994. By contrast, the reporting of egg-associated outbreaks did not start to decline until 1997, after the introduction of *S. enterica* ser. Enteritidis vaccination and flock hygiene program aimed at laying chicken flocks (*22*). This program included improved rodent control; feed monitoring; biohazard control; microbiological monitoring throughout all stages of production; and industry quality assurance schemes (*23*). This point also marks the start of the sharp decline in the human *S. enterica* ser. Enteritidis epidemic. Therefore, after considering the trends in the human and veterinary surveillance data and the findings of the 1988 case–control study (*7*), we infer that the epidemic in humans was associated with the consumption of both chicken and eggs. However, because control of *S. enterica* ser. Enteritidis in the production of chicken meat had much less effect on the course of the epidemic than control in eggs, we further conclude that the epidemic was largely attributable to the contamination of eggs. Persons became infected through the consumption of contaminated foods in commercial catering and home settings. The improvements in hygienic practice from egg production and distribution through the main supermarket chains has resulted in major improvements in the microbiologic quality of eggs bought by consumers in the United Kingdom (*20*).

Well-designed and -maintained national programs using hygiene control strategies to control *S. enterica* in primary production and distribution have also been successful in reducing the occurrence of *S. enterica* ser. Enteritidis in the food chain in the United States (*24*) and Denmark (*25*). However, data from harmonized surveillance of layer flocks in Europe indicate that *S. enterica* ser. Enteritidis infection remains a problem in egg production in many European Union member states (*26*). International surveillance data (*27*) and recently reported outbreaks also demonstrate that contamination of eggs remains a problem in many parts of Europe and the United States (*28*). This knowledge adds weight to our conclusion that the reduction in *S. enterica* ser. Enteritidis in chicken flocks in Great Britain stemmed from the introduction and maintenance of a suite of carefully designed and regulated interventions. In addition, accumulating evidence indicates that cross-sectoral national control strategies designed according to national needs and conditions can be extremely effective in reducing the risk to human populations worldwide. Our analyses of outbreak data show that the risks associated with the use of eggs in uncooked or lightly cooked desserts and sauces highlighted in previous studies (*4**–**7*) continued in England and Wales until *S. enterica* ser. Enteritidis had effectively been eradicated from egg production.

Analyses of the laboratory report and outbreak surveillance show that the overall impact of SE4 in England and Wales has been greatly reduced. However, control of non-SE4 has been less successful, a finding reflected in data from other countries in Europe (*29*). Investigation of outbreaks indicates that infection is mainly transmitted through the consumption of imported eggs in commercial catering (*12**,**30**,**31*). Outbreaks linked to desserts and sauces served in the catering sector have declined markedly, but Italian restaurants were commonly associated with egg-associated outbreaks during the epidemic stage (*31*). However, since 1997, only 4 non-SE4 and 1 SE4 outbreaks were associated with Italian restaurants (*31*). This change is thought to be because many restaurants switched to liquid pasteurized eggs or Great Britain–produced eggs for sauces and desserts. We performed an informal review of restaurant menus and found these desserts and sauces are still widely available in restaurants in the United Kingdom but that simple egg-based dishes served in Chinese restaurants still tend to be made using raw shell eggs (*31*). Previous research indicates that use of imported raw shell eggs and poor hygiene practice are more common in this sector (*31*). Therefore, a need exists for establishment of safer practices across the catering sector.

**Figure 6:**
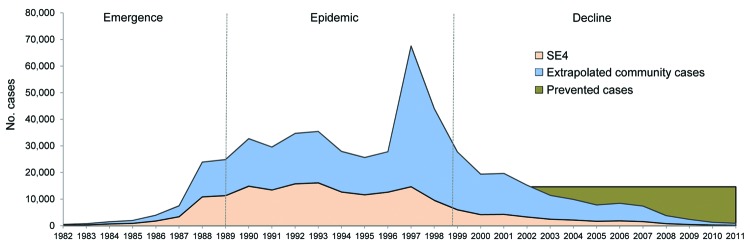
Trends in reporting of *Salmonella* *enterica* serovar Enteritidis phage type 4 (SE4), extrapolated burden of disease, and estimated number of cases prevented by SE4 elimination programs, England and Wales, 1982–2011.

Our estimates indicate that the *S. enterica* ser. Enteritidis epidemic in England and Wales had serious effects on the population and on the health care system. Had epidemic-stage infection levels been maintained from 1999 onward, we estimate that the introduction of effective interventions by the egg and poultry industries in Great Britain probably would have prevented ≈904,000 cases of illness in the community (225,973–20,247,145) (Figure 6), ≈6,300,000 days of illness, ≈26,000 hospitalizations, and ≈2,000 deaths since 1998. These figures should be treated with caution, but we suggest that a robust cost-benefit analysis of the epidemic and the interventions that led to its control would have great value for the development of improved food safety policies.
